# Temporal-thermal enhancement of porous cooking burners

**DOI:** 10.1038/s41598-024-82484-7

**Published:** 2024-12-28

**Authors:** Hossein Soltanian, Mohammad Zabetian Targhi, Mehdi Maerefat

**Affiliations:** Faculty of Mechanical Engineering, Tarbiat Modares University, P.O. Box 14115-143, Tehran, Iran

**Keywords:** Burner, Porous, Cooking period, Thermal efficiency, Firing rate, Engineering, Mechanical engineering

## Abstract

Porous combustion has drawn vast attention over the last few decades leading to a variety of progressing applications particularly in industrial kitchens and household appliances that require time sensitive heating. The present study experimentally investigates the relationship between cooking duration and the thermal efficiency of a cooking pot heated on a porous burner providing a valuable insights into the effectiveness of the heating process in terms of both time and fuel consumption. To facilitate this investigation, a dedicated test bench is designed and constructed, equipped with thermometers and timer to effectively monitor the temporal/thermal behavior of the heating process. A mixed temporal/thermal metric is utilized to evaluate a premixed natural gas fueled porous burner with a typical cooking firing rate (FR) ranging from 800 to 2050 kW/m^2^. The results show that the optimal burner-pot distance is D = 1.5 cm, minimizing the load-averaged time to thermal efficiency (TT) to 19.5 s. Compared to D = 3 cm with FR = 800 kW/m^2^, thermal efficiency improves by 5.5%, heating time shortens by 120 s, and average firing rates save 51 s overall. An open conventional burner is also explored to show that by shifting towards temporally promoted porous burners, up to 2 minutes of cooking time could be saved.

## Introduction

In recent decades, cooking burners have drawn significant attention due to their remarkable impact on energy efficiency and their contribution to global fuel consumption. In this regard, utilizing porous combustion could be a game changer in the cooking burners, and researchers have been exploring burner combustion and its associated heat transfer characteristics. However, the full potential of cooking burners in practical thermal systems has not been effectively harnessed with the pace of the cooking process being fully overlooked. Despite quite a few attempts on assessing thermal efficiency of cooking burners leveraging porous combustions, the studies in this area suffers from neglecting cooking time altogether which imposes a unilateral view leading to consumer dissatisfaction. The newly presented temporal insight highlights a prospect for future researchers and arises from a realistic concern aiming at a multi-faceted desirable cooking burner.

Numerous studies have sought to assess the thermal efficiency of employing porous media in burners as opposed to non-porous burners. As a primary investigation, Trimis and Durst^1^ established the necessary conditions for achieving stable combustion with a high level of thermal efficiency. Hashemi et al.^2^ explored flame stabilization in a dual-layer porous burner, examining heat efficiency and evaluating the impact of burner dimensions on it. Freddy J. Rojas et al.^3^ sought to enhance the thermal efficiency of industrial cookstoves by designing a burner that outperformed nationally available cookstoves using natural gas by averagely 5%. Deb et al.^4^ presented radiant porous burners designed for domestic LPG burners with a power range of 8–14 kW, demonstrating a thermal efficiency of 52%.

Soltanian et al.^5^ explored a porous cooking burner with thermal efficiency peaking at 29% in equivalence ratio of 0.99. they also showed that 70% of heat transfer to pot was conveyed through convection of pot bottom and pot side. Seung Wan Cho et al.^6^ conducted a study on optimizing the performance of a newly developed gas-fired radiant burner for a cooking pot. In their research they measured thermal efficiencies and found that as the firing rate increased, thermal efficiency decreased and conduction heat transfer became more dominant than radiation.

Jianyou Wang et al.^7^ examined heat transfer in domestic gas stove pots, finding that thermal efficiency is influenced by loading height and the addition of fins to the pot wall. Chih-Yung Wu et al.^8^ introduced a highly efficient flat flame burner for domestic stoves, outperforming traditional Bunsen flame burners in range and turndown ratio. Its efficiency and low pollution emissions remain stable regardless of the distance between the pot or pan, making it ideal for home applications. Comparing porous cooking burner in two modes of high and low thermal efficiency, Soltanian et al.^9^ provided design recommendation and investigated the operational condition as well as burner configuration in the journey from low to high efficient cooking porous burner. They found that over 15% of the total heat is gained from radiation in high efficiency mode. Some detailed literature scan should be brought to attention involving numerical and experimental investigations on pours combustion in domestic cooking applications:

Daniel Mulugeta Soma et al.^10^ studied on CFD analysis of porous medium burner which is made of two different sections used in order to improve the thermal efficiency and emission characteristics of domestic cooking stoves. Total six 3D models are considered for simulation and found that porous burner having center pipe diameter of 27.5 mm and 25 mm fluid inlet diameter gives the maximum surface temperature of 2250 K.

Saúl Laguillo et al.^11^ included CO emission analyses in their numerical studies and performed to provide a deeper insight into the CO formation and its relationship with the flame structure when natural gas is burnt in domestic gas cooking burners for increasing thermal efficiency. The influence of burner-to-pot distance, flame thermal power, primary aeration and inside-pot water temperature on CO emissions and thermal efficiency is evaluated. The analysis of the computational results reveals that CO emissions and thermal efficiency are strongly related to the relative boundary position of the inner premixed flame cone and the wall. A decrease in CO emissions is observed as primary aeration or wall temperature increases.

In this paper Shabani Nejad Hoda et al.^12^ numerically studied on combustion phenomenon and heat transfer in a three-dimensional rectangular porous radiant burner numerically. Methane-air mixture with five-step reaction mechanism is used to model the combustion process inside the porous matrix. Results show that by increasing the excess air ratio, porous burner operates under low maximum temperature and consequently less emission of pollutants in the combustion product.

Nageshwar Singh et al.^13^ present the experimental analysis of three types of porous burners and Liquified Petroleum Gas (LPG) was used. Burner was operated at different equivalence ratio ranging from 0.10 to 0.60. The maximum efficiency of 79% was observed for porous burner of diameter 90 mm at equivalence ratio of 0.49 and 0.84 kW power.

In this study Monikankana Sharma et al.^14^ investigate concept of Porous media combustion has been employed in a kerosene pressure stove and emissions. Further, the conditions for optimum efficiency and emission are brought out through a systematic. analysis with different burner geometry and exergy calculation. This research show the highest efficiency of the stove with porous media is found to be ~ 10% higher than the average thermal efficiencies of the stoves available in the Indian market.

Arwut Lapirattanakun et al.^15^ used a new Porous media burner Wasted Vegetable Oil (WVO) designed for cooking stove Appliances. DIN EN 203-1 testing standard was adopted and the experiment and Steam was successfully applied in a burner at this scale to atomize WVO droplet and entrain air into the combustion zone as well as to reduce soot and CO emission. Temperature, emissions, visible flame length, thermal efficiency as well as combustion efficiency were evaluated.

In this paper, V.K. Pantangi et al.^16^ deals with the performance tests of a PRB (porous radiant burner) used for LPG domestic cooking stoves. The burner consists of a two-layer porous media.For a given burner diameter, the performances of the burner, in terms of thermal efficiency and emission characteristics, are analyzed for different equivalence ratios and thermal loads (wattages).The maximum thermal efficiency of the LPG cooking stoves with a PRB was found to be 68% which is 3% higher than that of the maximum thermal efficiency of the conventional domestic LPG cooking stoves.

Chih-Yung Wu et al.^17^ applied a clean and highly efficient domestic stove burner composed of a flat flame burner for cooking and water heating. The flame appearance, temperature distribution, relative thermal efficiency and pollution emissions in terms of Emission Index of CO (EICO) and Emission Index of NOx (EINOx) were measured and analyzed. The results show that the operating range, turndown ratio, and pollution emissions of the flat flame burners are superior to those of traditional Bunsen flame burners.

Freddy J. Rojas et al.^18^ research improve the thermal performance of industrial cookstoves using natural gas in Metropolitan Lima to promote good use of energy and reduce the impacts of climate change. The burner designed and manufactured in previous research gave a wide range of natural gas power according to the working pressure used and high efficiency of approximately 57% compared to the cookstoves that are sold nationwide, which on average is 53%. The advantage of this prototype cookstove is that it uses operating pressures between 23 and 34 mbar compared to stoves in the Peruvian market that do not have a standard of the maximum gas pressure.

In this paper Jianyou Wang et al.^19^ studied on heat transfer capacity of the pot in domestic gas stoves numerically and experimentally and results show that with the increase in loading height Z, the thermal efficiency decreases After adding fins to the wall, it is found that the thermal efficiency increases with an increase in fin height H. By considering the total mass of the pot and thermal efficiency, we select the fin number *N* = 42, and its thermal efficiency is increased by 7.12% compared with the original pot. Experimental results confirm that the increase is 8.2% under the same conditions. Moreover, it is shown that the thermal efficiency at first increases to a maximum value then decreases with the augment of inclination angle.

Prasad Boggavarapu et al.^20^ work on the thermal efficiency of a conventional domestic burner both experimentally and numerically for liquefied petroleum gas and piped natural gas fuels. Experiments show an improvement in burner thermal efficiency of 2.5% for LPG with the modified design, and 10% for PNG with the optimal loading height, demonstrating that the CFD modeling approach developed in the present work is a useful tool to study domestic burners.

In the context of thermal cooking systems, the significance of considering heating time is crucial, yet it has not been adequately addressed. This study seeks to address this gap by examining the heating duration of a pot using a porous burner and presenting a unified metric that captures both its temporal and thermal efficiency. Heating time interval is to be considered in order to promote the cooking system and effectively present a novel dual-faceted solution for cooking burners. In the present research and for the first time a mixed temporal/termal metric is introduced and utilized to evaluate a premixed natural gas fueled porous burner with a typical cooking firing rate. provide guidance for cooking burner design and optimization. The present approach has the potential to be generalized to other time-sensitive thermal systems across a broader range of FR.

## Experimental setup

**Fig. 1:**
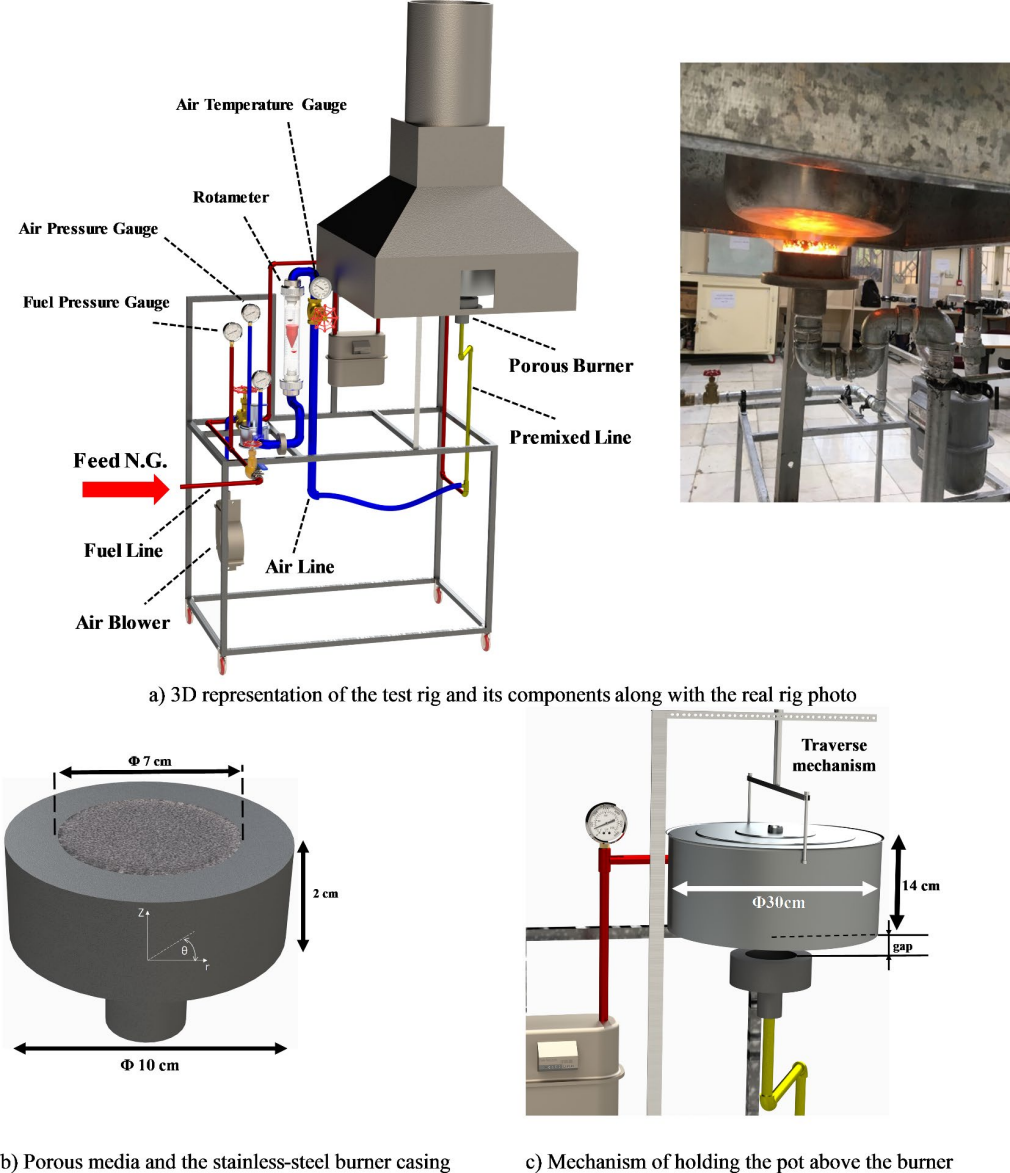
Overall view of the test rig (**a**), details of porous burner (**b**) and mechanism of holding the pot above the burner with pot dimensions (**c**).

The experimental test rig for the burner-pot system was designed and constructed for natural gas, with a cooking firing rate ranging from 797 to 2043 kW/m^2^, using SiC as the porous ceramic material. All tests were conducted under ambient pressure and temperature conditions, specifically at 88.5 kPa and 26.8 °C. Figure 1a shows the complete setup, highlighting the main components involved. The placement of the round SiC porous media, with 85% porosity (20 ppi) and an average pore diameter of 1.5 mm, inside the stainless-steel burner casing, along with its dimensions, is depicted in Fig. 1b. Furthermore, Fig. 1c provides a close-up view of the test conditions, illustrating the integration of the cooking pot within the setup and offering detailed information.

## Theoretical framework

A test rig was designed and built to experimentally investigate the time duration and energy transfer in a cooking porous burner, aiming to compare its performance in terms of temporal efficiency and thermal efficiency. The tests were performed according to Iranian standard ISIRI-10,325^21^ which is itself driven from European standard of BS EN 30-2-1:1998^22^. Thermal efficiency for a cooking burner is popular to be obtained by Eq. (1)^21^:

1 $$\:{\eta\:}_{th}=\frac{({m}_{w\:}{C}_{w}+{m}_{p}{C}_{p})\times\:({T}_{2}-{T}_{1})}{{V}_{f}LHV}$$

The majority of researchers have utilized Eq. (1) to evaluate thermal efficiency^4^ where $\:{m}_{w\:}$and $\:{m}_{p}$, $\:{C}_{w}$, $\:{C}_{p}$, $\:{T}_{1}$ and $\:{T}_{2}$ stands for the mass of water, the mass of pot, the specific heat capacity of water, the specific heat capacity of pot, initial temperature, and final temperature. $\:{V}_{f}$ represents the volume of fuel consumed during the heating process and finally, $\:LHV$ is the lower heating value of natural gas which according to ISIRI – 10,325 (under test conditions at atmospheric pressure and a temperature of 15 ^o^C) is 37.78 MJ/m^3^. Firing rate is obtained from Eq. (2):

2 $$\:FR=\frac{LHV\times\:Q}{A}$$

In which $\:FR$ stands for firing rate, $\:LHV$ as mentioned is lower heating value of fuel, $\:Q$ is volumetric flow rate of the fuel and $\:A$ denotes the burner area associated with the porous media surface. The pot is made of aluminum, with a diameter of 28 cm and a height of 15 cm as recommended by standard^10^. In the present study, a newly proposed metric is introduced to incorporate both heating time and thermal efficiency, as defined by Eq. (3):

3 $$\:TT=\frac{\varDelta\:t}{\eta\:{}_{th}}$$

Where TT represents time to thermal efficiency and $\:\varDelta\:t$ stands for the duration of the heating period. A lower value of this proposed index indicates a more efficient heating process.

## Results and discussion

**Fig. 2:**
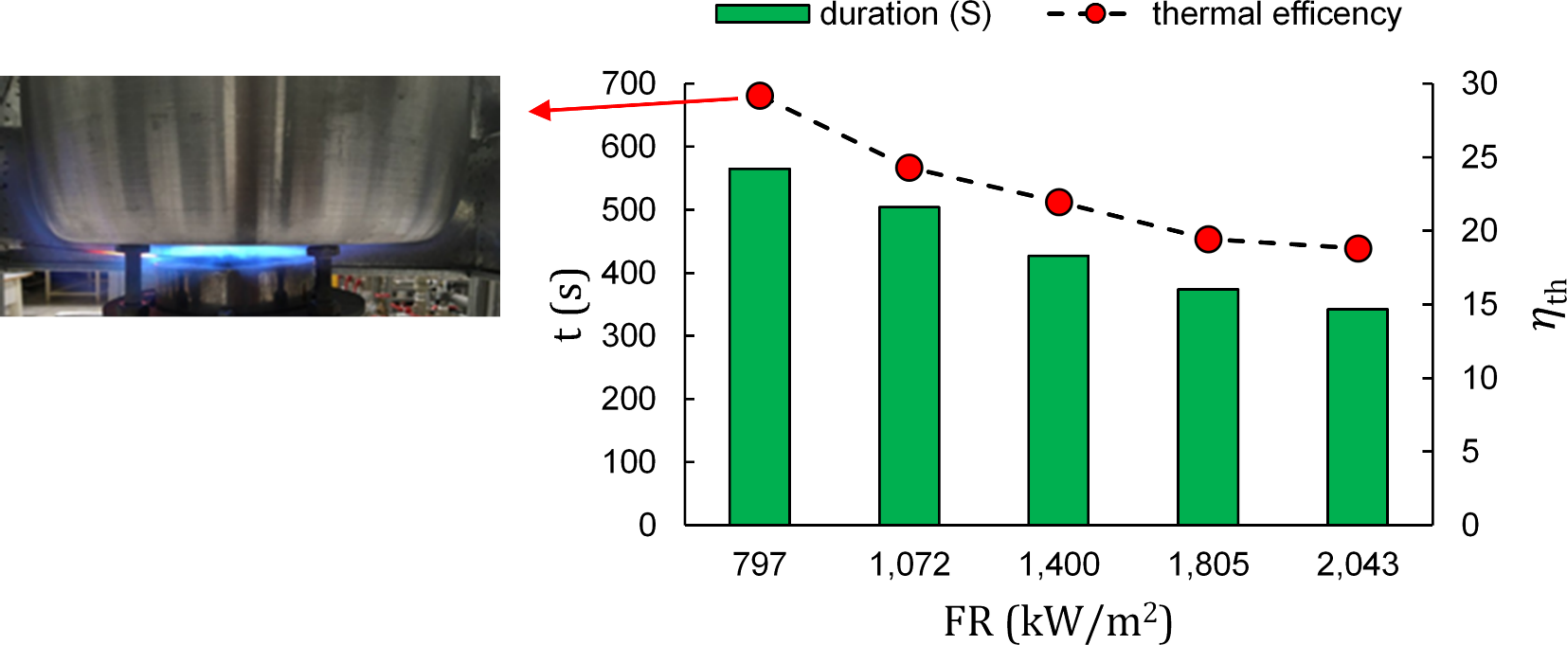
Duration of water heating and thermal efficiency relative to operating firing rates in porous burner (D = 1.5 cm)

To examine the duration of the heating process, the time taken to heat water from an initial temperature of 25 °C to a final temperature of 90 °C, as outlined in^21,22^, is presented alongside the thermal efficiency for D = 1.5 cm in Fig. 2. It is important to note that the firing rates are adjusted to achieve the highest thermal efficiency, with the equivalence ratio falling within the range of 0.9 to 1.0 in all cases. Taking into account the burner surface area, the lower heating value of natural gas, and the feeding volumetric flow rate, the thermal efficiency ($\:\eta\:{}_{th}$) has an uncertainty of 0.25%. Additionally, the heating time span is measured with an error margin of 1 s in the subsequent results.

**Fig. 3:**
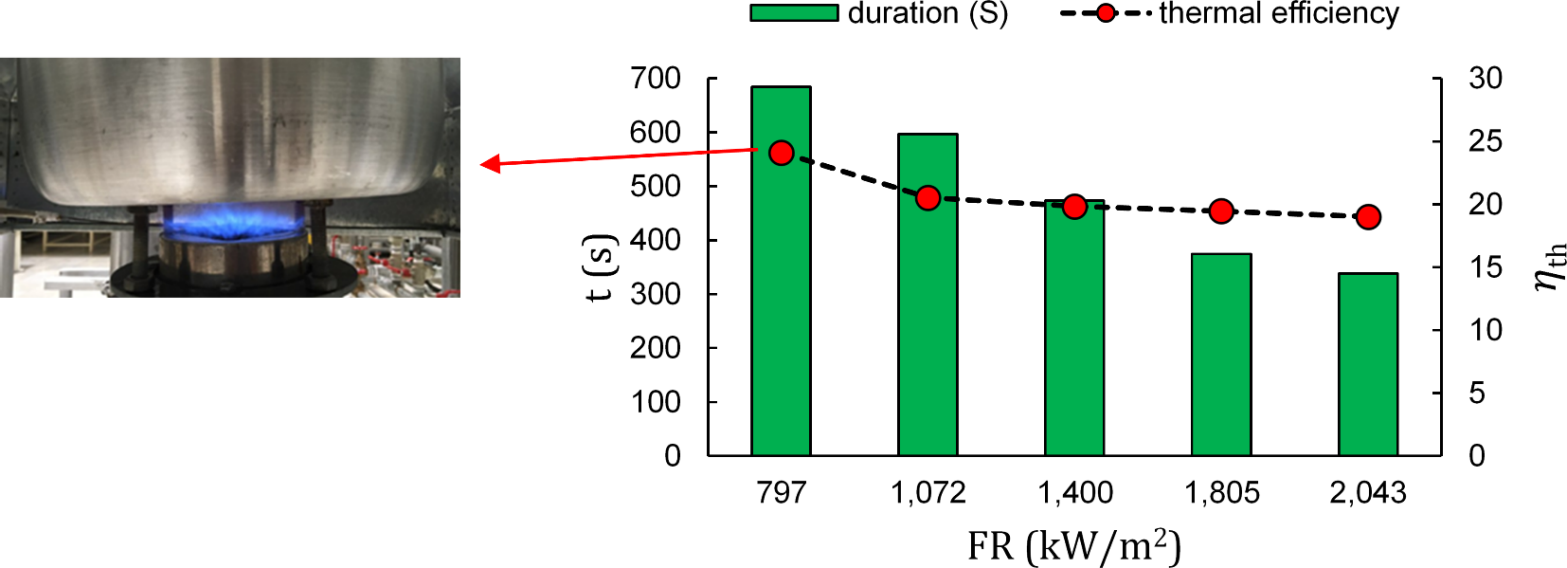
Duration of water heating and thermal efficiency relative to operating firing rates in porous burner (D = 3 cm).

Figure 2 suggests that an increase in input firing rate results in a more efficient and faster heating of the water. Conversely, thermal efficiency declines, raising concerns about fuel misconsumption. The same result is noticeably observed in the second standard gap between pot and burner, D = 3 cm, which is illustrated in Fig. 3. The opposite trends regarding thermal efficiency and heating time indicate the necessity of using a comprehensive index for these two variables. Actual flame photos are provided for both distance gaps in Figs. 2 and 3 to better assist in heating time and thermal efficiency difference explanation.

From thermal efficiency standpoint, at the initial firing rate ,797 kW/m^2^, Fig. 2 (D = 1.5 cm) shows an evident brighter flame than Fig. 3, which enhances the radiant heat transfer rate. This improvement results in a 5% increase in thermal efficiency compared to the configuration with a gap of 3 cm shown in Fig. 3.

Temporally speaking, contrary to Fig. 3, in Fig. 2a side flame is developed on the pot side creating a slim while attached area of hot gas on the pot side which is the main contributor to reducing heating time. Due to such a flame structure heating duration has dropped from 684 s for D = 3 cm (Fig. 3) to 565 s for D = 1.5 cm (Fig. 2) which highlights 2 min of time saving in cooking.

Based on the distance gaps in Figs. 2 and 3, although thermal efficiency at higher firing rates appears to be less influenced by the firing rate, the heating duration does not follow the same pattern and continues to decrease significantly meaning that steady state heating time falls behind that of thermal efficiency especially in case of D = 3 cm.

**Fig. 4:**
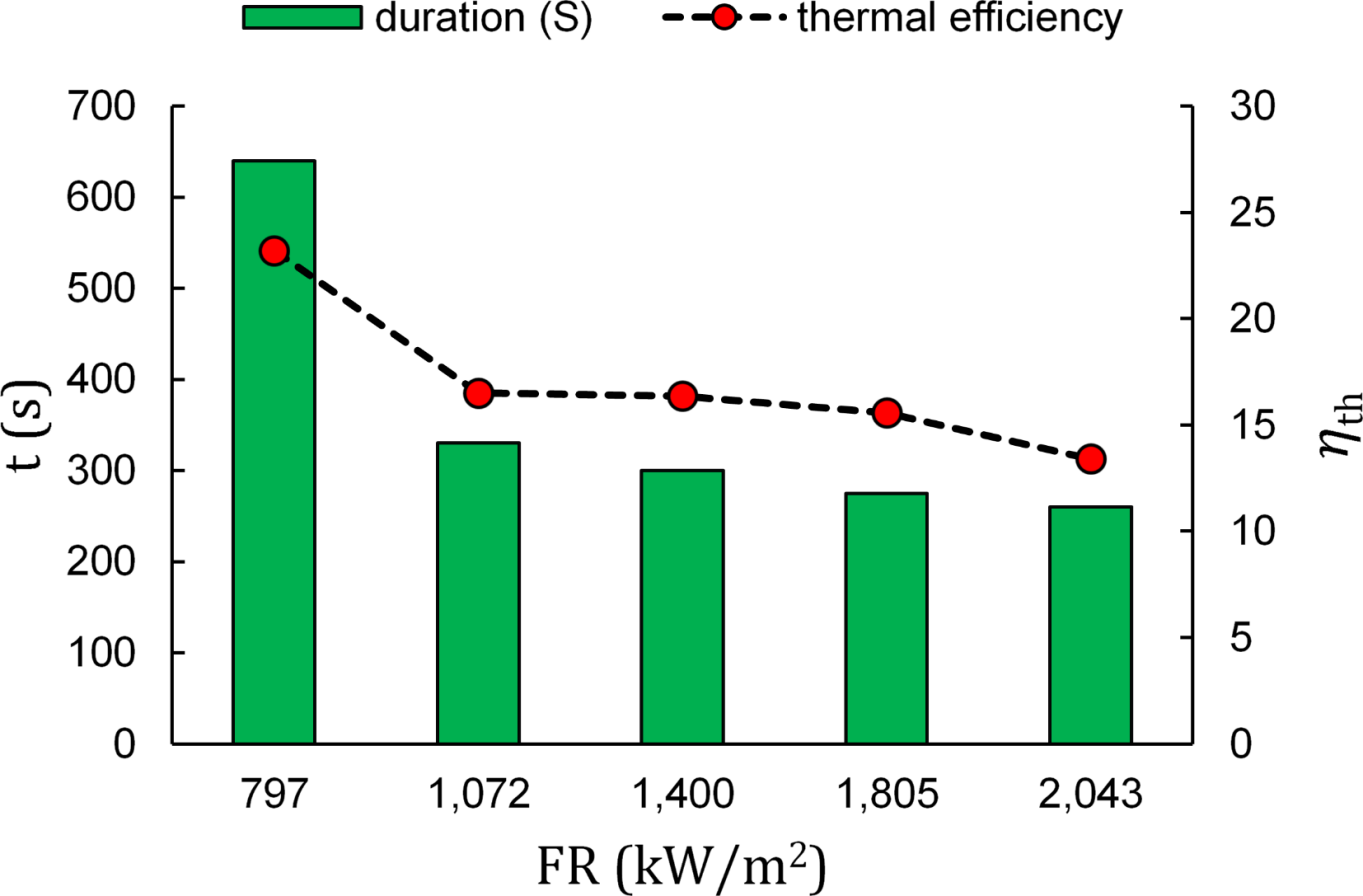
Duration of water heating relevant to thermal efficiency in operating input firing rates in a conventional non porous burner (D = 1.5 cm).

Replacing the porous burner with a simple open flame conventional burner provides a valuable insight into how porous combustion benefits the burner from time and thermal efficiency viewpoints. To this end, Fig. 4 shows the similar graph for a conventional non-porous burner.

An abrupt decline in heating time is observed after the first FR which stems from the inability to retain hot gas momentum once the flame reaches the bottom surface of the pot. As a result, in the first FR the flame cannot reach the side of the pot and negatively affects heating time. This in turn affirms the role of attaching side flame on heating time. Subsequently, by increasing input FR the flame is developed firstly on pot bottom and later on, on sides. However thermal efficiency value ($\:\eta\:{}_{ave}=$17.0) is still incapable of reaching that of porous burners ($\:\eta\:{}_{ave}=$22.7) due to porous combustion advantages (including naturally preheated, well distributed mixture resulting in a more stable, radiative and pot attached flame).

The average heating duration, thermal efficiency, and the resulting TT index for the two gap distances are presented in Table 1, allowing for a comparison with conventional non-porous burners.

**Table 1:** Averaged parameters across operating firing rates for porous and non-porous burner.

Burner layout	Δt_ave_	η_ave_	TT_ave_
Porous D = 1.5 cm	442.4 S	22.742%	19.4530 S
Porous D = 3 cm	493.2 S	20.583%	23.9615 S
Non porous D = 1.5 cm	361 S	17.006%	21.2278 S

As can be seen in Table 1, the shorter heating time of the conventional burner, Δt_ave_, comes at the cost of drastic energy loss as its thermal efficiency considerably low. Comparing the average TT values indicates that the optimal trade-off between heating speed and energy efficiency favors the porous burner with a small distance gap of D = 1.5 cm, achieving a TT_ave_ average of 19.453. Therefore, despite occasionally having shorter heating times, the conventional burner is not a suitable solution for cooking purposes.

With the optimal distance gap determined to be D = 1.5 cm, the next step in refining the burner design is to identify the ideal firing rate for the heating process. Table 2 entails TT values for the operating firing rates.

**Table 2:** TT values for the proper distance of D = 1.5 cm across the operating firing rates.

Firing rate (kW/m^2^)	797	1072	1400	1805	2043
TT (S)	19.3493	20.74074	19.4444	19.2288	18.1915

**Fig. 5:**
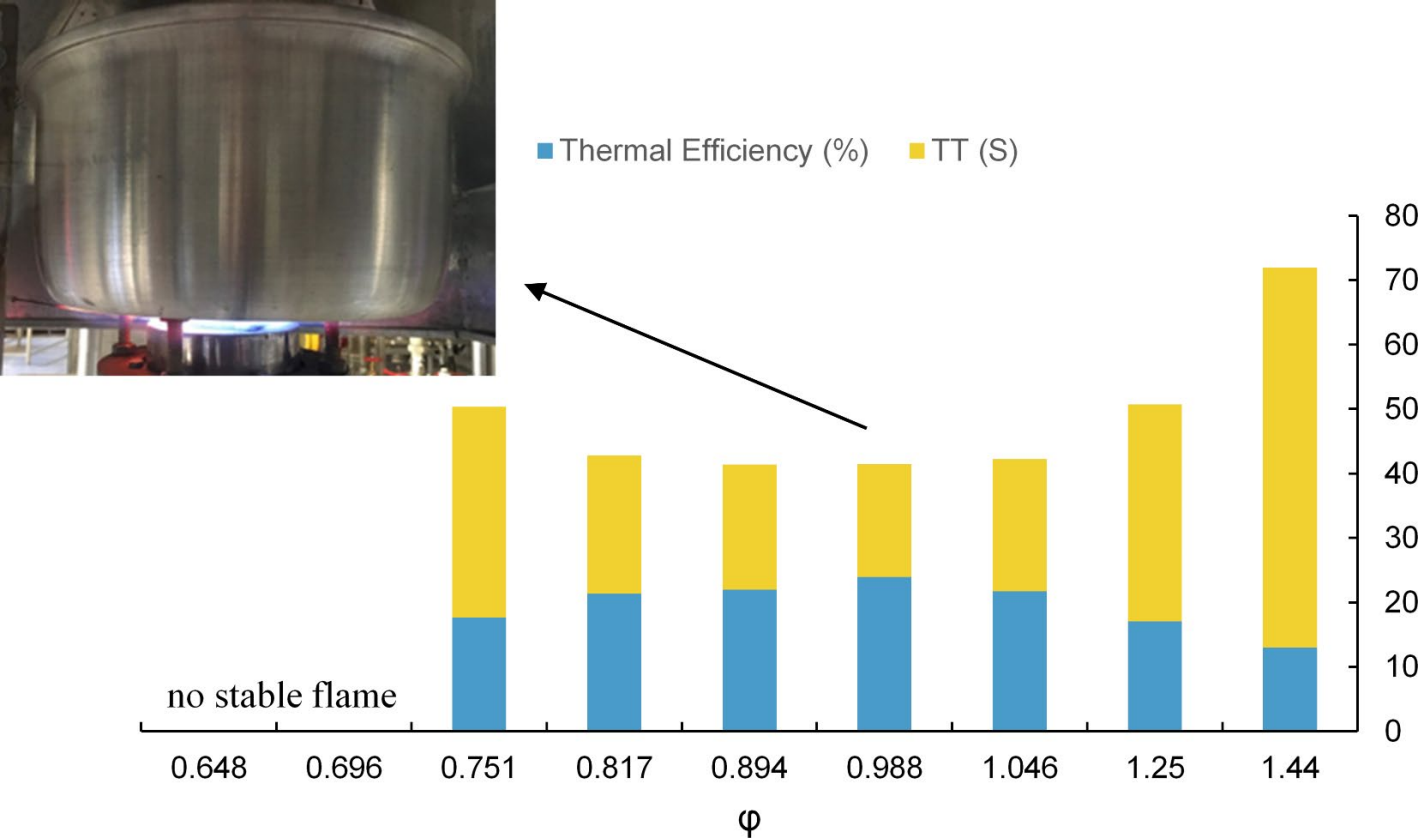
Thermal efficiency and TT index with respect to equivalence ratio for the firing rate of 2043 kW/m^2^.

Based on Table 2, it can be concluded that the most suitable firing rate for the porous burner at a distance of D = 1.5 cm is 2043 kW/m^2^, achieving the lowest TT value of 18.19. This identifies the optimal condition of D = 1.5 cm and FR = 2043 kW/m^2^ for the best performance. This optimized design point should be finalized by a subsequent equivalence ratio for the fixed FR = 2043 kW/m^2^ which is presented in Fig. 5.

As evident from Fig. 5, the optimal φ is achieved at 0.988, showcasing maximum thermal efficiency ,23.93%, and minimum TT ,17.51 S, simultaneously. Deviating towards higher or lower equivalence ratios leads to the appearance of a highly dethatched-uncontrolled side flame or the complete absence of a side flame, respectively. These two adverse effects diminish as the equivalence ratio approaches mid-range values, benefiting from a radiant, attached flame that maximizes fuel efficiency and achieves the shortest heating time (at φ = 0.988).

## Design and operation guide for high efficiency porous burner

Burner operating condition together with its configuration plays a crucial role in suitable and effective usage of them in heating up the target quick and energy effective. As a result and in order to reduce cooking time without sacrificing thermal efficiency, its distance from burner and the proper firing rate and equivalence ratio should be meticulously adjusted.

The attainment of high efficiency in porous burners with previously demonstrated advantages is influenced by the following conditions:

- Adjusting the distance between porous burner surface and the target pot has a crucial role in enhancing both temporal and thermal heating efficiency.
- Setting the input power in a suitable range to make the combustion attached to the surface to enhance thermal efficiency and yet maintaining the side flame to keep the process fast enough.
- Picking the proper equivalence ratio to obtain a radiant attached flame resulting in a higher thermal efficiency and letting this flame on the side of pot to get a proper temporal efficiency.
- Forcing the flame to attach to pot side with a narrow depth is beneficial to lowering heating time.
- Preheating the mixture using wire mesh and three arrays of alumina balls.
- Getting the side flame as radiant yellowish as possible by adjusting input load and equivalence ratio to leverage side radiative heat transfer for minimizing heating time.
- Preheating the mixture using wire mesh and arrays of alumina bulls along with using proper mixing and distribution to make combustion in the porous media and its surface uniform.

## Conclusion

This study highlights the effectiveness of optimizing porous burners for enhanced thermal and temporal efficiency. A burner-pot distance of 1.5 cm and a firing rate of 2043 kW/m^2^, combined with an equivalence ratio of 0.988, resulted in significant reductions in cooking time and improvements in energy efficiency. The findings emphasize the role of radiant and attached flames in minimizing heating durations, providing insights into the potential of porous burners to replace conventional designs for time-sensitive applications. Such optimizations not only improve performance but also contribute to energy savings and operational cost efficiency in domestic and industrial contexts. The key findings include:

- With appropriate preparations such as making the flame radiant and attached to pot side with little thickness, heating duration could drop from 640 S in an ordinary burner to 342 S (up to 47% quicker heating).
- Joint fast heating with high thermal efficiency depends on radiative, thin and attached flame and introduces side flame as the main contributor to short heating time.
- For porous burner setting the distance to 1.5 cm rather than 3 cm increases the thermal efficiency by up to 5.5% and lowers heating duration to 120 S for firing rate of 800 kW/m^2^. On an average based firing rates, it is 51 s of time saving.
- By migrating from ordinary cooking burners to the ultimately designed one presented in this study (in terms of distance gap, operational input power, equivalence, thermal and temporal efficiency), could lead to 0.15 m^3^ saving of fuel per each roughly 8-minute use (Total annual usage is 112 m^3^^23^). This is equal to 165$ per year for each single cooking burner.
- The archived improvement of the duration by 2 min together with 5.5% of thermal efficiency for a typical emission index in a natural gas burner (CO: 0.1–0.4 g and NOx: 1.5–2.5 g per m³ of natural gas) results in a noticeable reduction of 13.5 and 90 g of CO and NOx, respectively.
- To speed up heating and effective fuel consumption to lower its costs, it is recommended to use porous burners over traditional ones as it lowers the TT index from 22.2 S to 19.4 S in a similar pot-burner distance.
- In low FR = 800 kW/m^2^, Porous burner shows a noticeable priority over conventional burner from both temporal and fuel consumption perspective, lowering heating duration from 640 S to 565 S and thermal efficiency from 23.2 to 29.2%.
- The input firing rate is cooking oriented but could be easily generalized to accommodate any time sensitive burner and application.
- The design point for the cooking porous burner is reached to be D = 1.5 cm, FR = 2043 kW/m^2^ and Ф=0.98 offering heating time duration of 342 S with TT = 18.19 S.
- Departing from ordinary porous burners to temporally promoted porous burners in cooking stoves, could save up to 2 min of cooking time from 25 °C to 90 °C.
- For the best FR and D, a broad range of 0.65 < φ < 1.44 showed the least TT of 17.51 S occurring at φ = 0.988 featuring the most radiant-attached flame to bottom and sides of the pot.

The input thermal powering rate could impose some limitations on the study along with the precises content of natural gas which varies slightly throuought the year. However, Future research should aim to validate these results across diverse cooking scenarios and explore their applicability to other burner designs, fuels, and configurations. Developing advanced materials for the porous medium to further enhance flame radiation and its thermal and temporal efficiency while maintaning durability is also forseen.

## Data Availability

The authors declare that the data supporting the findings of this study are available within the paper. Should any raw data files be needed in another format they are available from the corresponding author upon reasonable request. Source data are provided with this paper.

## List of symbols

A: Area (m^2^)
C: Specific heat (J kg^− 1^K^− 1^)
D: Distance gap between pot and burner (cm)
FR: Firing rate (kWm^− 2^)
LHV: Lower heating value (MJm^− 3^)
m: Mass (kg)
ppi: Pore per inch
Q: Volumetric flow rate (m^3^S^− 1^)
T: Temperature (K)
TT: Time to thermal efficiency (S)
V: Volume (m^3^)
η: Thermal efficiency
Δt: Cooking duration (S)
φ: Equivalence ratio
